# Terminal anorexia nervosa: three cases and proposed clinical characteristics

**DOI:** 10.1186/s40337-022-00548-3

**Published:** 2022-02-15

**Authors:** Jennifer L. Gaudiani, Alyssa Bogetz, Joel Yager

**Affiliations:** 1 CEDS-S, FAED, Gaudiani Clinic, Denver, CO USA; 2 Department of Psychiatry, University of Colorado School of Medicine, Aurora, CO USA

**Keywords:** Anorexia nervosa, Case presentation, Criteria, Definition, Hospice, Medical aid in dying, Obsessive compulsive disorder, Palliative, Severe and enduring anorexia nervosa, Terminal

## Abstract

**Background:**

Most individuals with eating disorders will either recover, settle into an unrecovered but self-defined acceptable quality of life, or continue to cycle from crisis to relative stability over time. However, a minority of those with severe and enduring eating disorders recognize after years of trying that recovery remains elusive, and further treatment seems both futile and harmful. No level of harm reduction proves achievable or adequately ameliorates their suffering. In this subgroup, many of those with anorexia nervosa will experience the medical consequences of malnutrition as their future cause of death. Whereas anyone who wishes to keep striving for recovery despite exhaustion and depletion should wholeheartedly be supported in doing so, some patients simply cannot continue to fight. They recognize that death from anorexia nervosa, while perhaps not welcome, will be inevitable. Unfortunately, these patients and their carers often receive minimal support from eating disorders health professionals who are conflicted about terminal care, and who are hampered and limited by the paucity of literature on end-of-life care for those with anorexia nervosa.

**Case presentation:**

Three case studies elucidate this condition. One patient was so passionate about this topic that she asked to be a posthumous co-author of this paper.

**Conclusions:**

Consistent with literature on managing terminal illness, this article proposes clinical characteristics of patients who may be considered to have a terminal eating disorder: diagnosis of anorexia nervosa, older age (e.g. age over 30), previous participation in high quality care, and clear and consistent determination by a patient who possesses decision-making capacity that additional treatment would be futile, knowing their actions will result in death. By proposing the clinical characteristics of terminal anorexia nervosa, we hope to educate, inspire compassion, and help providers properly assess these patients and provide appropriate care. We hope that this proposal stimulates further expert consensus definitions and clinical guidelines for management of this population. In our view, these patients deserve the same attendant care and rights as all other patients with terminal illness, up to and including medical aid in dying in jurisdictions where such care is legal.

## Background

As a patient with severe and enduring anorexia nervosa advocating for my legal right to MAID (medical aid in dying), I confronted numerous obstacles and challenges from the medical profession, related not just to the question of whether I should have access to MAID generally, but more so, how my anorexia, a psychiatric condition frequently misunderstood by the medical community, interacted with my decision making capacity and desire to pursue MAID as one potential option knowing that my illness was indeed terminal.

–Alyssa

The vast majority of potentially terminal illnesses carry with them thoughtfully considered and evidence-based staging criteria. These criteria allow patients and clinicians to distinguish mild and likely curable presentations of the disease from irreversible, pre-terminal and terminal stages. Medical specialties treating cancer, organ failure, or various infectious diseases have dedicated considerable attention and resources to delineating levels of severity. While preliminary suggestions for labeling severe and enduring anorexia nervosa (SE-AN) [1] and for staging the disorder have been proposed [2], generally accepted staging criteria for anorexia nervosa (AN) have not yet been developed. Remarkably, the same diagnostic label (“AN”) and treatment criteria that apply to teenagers only a few months into their disorder are also used for patients who are decades older, who have lived through innumerable admissions to inpatient and residential care facilities, and whose quality of life has been irrevocably damaged by persistent, severe mental and physical illness. The field acknowledges SE-AN as a somewhat distinct clinical condition, but despite thoughtful clinical and research efforts [3, 4] the designation has not been formalized as a diagnosis, and consensus regarding criteria for SE-AN remains elusive [5].

AN carries the second highest mortality rate in the DSM-5 after opioid use disorder, with a death rate estimated at 5–16 times that of the general population. [6, 7] Several important recent studies confirm and expand upon these data. A specialized medical inpatient unit in France for those with severe anorexia nervosa evaluated 384 patients admitted over 17 years, with a mean age at admission of 29.4 years old. The standardized mortality ratio (SMR) was 15.9 for women and 22.4 for men, where older age was determined to be a major predictor of mortality. The mean age at death was 41.3 (± 15.3) years, on average two years after hospital admission. The SMR was maximally increased for patients whose first admission to the unit took place while they were between 25 and 35 years old. Specifically, those admitted between 30–34 years old had the highest SMR of 26. Somatic (medical) causes accounted for 43% of deaths, while 11.5% of deaths were caused by suicide. [6] In a registry-based observational epidemiological study encompassing the entire population of Denmark over 44 years, the SMR for all-cause mortality reached a maximum of about 6 in the age group 20–34 years, and the SMR for suicide in those with AN was 11. Natural causes accounted for two-thirds of death in those with AN. [8] Finally, a retrospective cohort study evaluated 19,041 individuals with an eating disorder in Ontario, Canada, using administrative healthcare data. The entire cohort, not comprised only of those with AN, had an SMR of 5; they found that potential years of life lost were 6 times higher than expected compared with the Ontario population. Similar to the other studies, peak values for SMRs were observed among adults between 30 and 44 years old, and again the SMRs observed in males were almost two-fold higher than in females. [9] Importantly, the profound suffering inherent in AN drives the high suicide rate noted in multiple studies, where up to 20% of patients who die prematurely do so by suicide [10]. Compared with gender- and age-matched groups, patients with AN are 18 times more likely to die by suicide [11].

Based on these data, AN can unquestionably prove fatal. Despite this fact, the field lacks clinical roadmaps for compassionate, appropriate care for those who will not be able to survive. This does great disservice to patients and their families. By comparison, we do not expect individuals with metastatic lung cancer who have disease progression despite past treatments, which often come with negative sequelae, to keep presenting for those same ineffective treatments. Rather, they are more likely to receive the psychological preparation, connection, and medical and emotional support offered to patients with terminal conditions. Although current laboratory measures and imaging studies by themselves are unable to help us stage patients with AN, based primarily on clinical histories and patients’ narratives we can better understand the clinical course of this illness and the subset of patients with AN who may seek palliative care [12–14].

There is growing recognition that palliative care may be appropriate for some patients, but the clinical characteristics for *terminal anorexia nervosa* have not been proposed. Delineating and validating this stage would greatly assist patients, families, and clinicians across disciplines, especially those in palliative and hospice care. Designating terminal AN may more readily enable patients to receive palliative care, hospice care, and emotional and practical resources for loved ones, as well as access to medical aid in dying (MAID) where legal. Therapeutic goals in these situations are to ameliorate suffering and honor the life lived. Of note, MAID is offered to individuals whose death is inevitable within six months from an underlying disease process; it provides patients a choice in *how* they die, not *whether* they die. It is not a means of suicide.

In this paper, we describe three cases of exceptional people whose AN was terminal, and who died peacefully with family around them. All three were patients of a private practice outpatient medical clinic specializing in eating disorders in Denver, Colorado. One patient, who had been a medical researcher herself, was so passionate about the topic that she asked to join as a posthumous author on this paper so her voice could be heard. The other two patients’ parents consented to share their son’s/daughter’s stories and reviewed, and all three families edited the relevant story prior to manuscript submission. All families agreed that first names should be used instead of a pseudonym or initials in order to emphasize the truly personal, real-life origin of these stories. Based on these experiences and others [12, 14], we conclude by proposing a set of clinical characteristics of those who can be identified as having terminal AN.

## Case presentations

### Case 1: Aaron

Aaron was a 33-year-old man with a long history of restrictive AN, severe obsessive compulsive disorder (OCD), recurrent major depression, and chronic suicidality. He had been a sensitive child with low self-esteem and perfectionism from a young age. His parents noticed OCD traits from early childhood, but he did not receive this formal diagnosis until years later.

During his freshman year in high school, a health class warned about the risks of “eating junk food.” Aaron began to run regularly and played hours of basketball daily. One by one, he eliminated dietary fats and created food rules. His parents thought this was just a stage, an assessment they came to understand very differently over time, but they eventually recognized his serious problems and established a treatment team. Later in high school, Aaron was hospitalized several times for AN, participated in family therapy, and required his mother’s presence, even at school, to complete meals. Despite graduating as valedictorian of his high school class, he was initially too ill to start college. Later, his attempt to begin college was thwarted by his need for constant supervision of food intake. Aaron’s perfectionism and self-criticism ultimately ended his college career.

Over the next two decades, Aaron spent countless months in medical hospitals and in inpatient and residential eating disorder settings. He repeatedly gained the weight required for discharge so that he could return home, only to inevitably relapse. He felt mortified and guilty about the amount of money his family spent on his treatment, and he was acutely aware of life passing him by.

In his early 30 s, following a long and serious downward spiral during which he refused a higher level of care, his family finally threatened to call 911 if he did not enter treatment. Consequently, he was admitted to an inpatient eating disorder program. After first spending time in a hospital setting for stabilization where he refused to eat, a feeding tube was placed. A court mandate to ensure ongoing treatment was requested and granted on grounds of grave disability from his mental illness, and he spent the next 10 months against his will in inpatient and residential eating disorder care. He cut off communication with his parents but allowed the treatment team to talk with them. Eventually his therapist convinced him to have family sessions over the phone; it was the only time his parents could talk to him.

Aaron’s persistent resistance to treatment throughout his stay at the eating disorder program caused difficulty in maintaining his nutritional stability. He underwent in-depth exposure and response prevention therapy around food as he continued to be tube fed, and he was finally able to sustain his weight with oral food. At that time, he only agreed to eat to avoid the prospect of being administered olanzapine against his will, as he feared this medication would cause him to gain weight.

After Aaron had been fully weight restored for several months, he was stepped down to a partial hospital program (PHP). He immediately restricted intake and proceeded to lose nearly a pound a day, resulting in readmission to residential treatment where, after intensive efforts, he once again achieved his target weight. Aaron struggled with basic activities of daily living due to his OCD. For instance, he resisted using lotion or lip balm as he feared they might be absorbed into his skin as calories. He completed a course of intranasal ketamine in hopes of alleviating his OCD, depression, and suicidality, but ketamine treatments had no meaningful impact.

Author JG (hereafter referred to as “Dr. G”) first met Aaron for an outpatient medical consultation after he had completed this most recent residential treatment and was once again about to step down to PHP. This consultation constituted one component of an organized, comprehensive future discharge plan. In this initial medical visit, after a year of residential treatment, Aaron was medically stable. He desperately missed his eating disorder behaviors, fantasized about eating less and losing weight, and wished his AN would have already taken his life. Aaron mused that his all-or-nothing, perfectionistic temperament made the unknown terrifying, but he felt proud of how rigidly he had previously adhered to his eating disorder rituals, as he believed that few others could achieve a similar degree of calorie restriction. Despite his long history of treatments, including his year of suffering through the long court-mandated treatment, Aaron had never meaningfully changed his eating related attitudes, thoughts, or behaviors. He had absolutely no motivation for recovery.

During the initial consultation, Dr. G informed both Aaron and his emotionally supportive and highly invested parents that she could offer ongoing outpatient medical care along one of two pathways. In one, Aaron would complete PHP, be discharged to home (where he lived with his parents), see a therapist and dietitian regularly, and work on whatever degree of recovery he could bear, aiming toward a quality of life that he called “living productively.” Should he decide that he required a higher level of care, the team would promptly support that choice. On the second pathway, if Aaron relapsed and declined readmission, the outpatient team would no longer battle with him to seek a higher level of care, given the futility of his most recent, autonomy-depriving treatment course. Rather, the family and team would support and comfort him until such time as he required home palliative care and eventually hospice support. Aaron initially felt that these two choices were needlessly stark and binary, and he settled back into PHP.

Two months after the initial consultation, Aaron continued to endure PHP, primarily to honor his commitment to his residential treatment team that he would see through his course of treatment. But he felt no better. He received a course of intravenous ketamine to supplement the intranasal ketamine treatment initiated in the residential program, but he experienced no improvements in mood, hopelessness, or OCD. Just before discharge to his parents’ home, he still hadn’t decided which treatment pathway to choose. He would not accept psychiatric medications, and a team consisting of a physician, therapist, and dietitian was established to care for him at home.

However, starting on the day of his discharge from PHP, Aaron stopped eating altogether, a course of behavior that is rare even in those with severe AN. He drank only water, stating, “I don’t want to die, but my eating disorder is in charge.” A week later, he met with his longstanding outpatient therapist. She was very apprehensive about his ability to remain in the community, and she felt ambivalent concerning what her role might be if he insisted on remaining at home. Aaron told her, “I wish I could eat, but I won't eat; I don't want to die, but I feel hopeless that there's any other pathway.” The therapist worried that she would be forced to have Aaron detained against his will in his home state. A formal decision-making evaluation was performed by a local psychiatrist, and Aaron was found to possess decisional capacity. Consulting with his home therapist, Dr. G proposed that Aaron’s refusal to eat was less about “wanting to die” than simply accepting that he could not live—he was not “attracted to life” [17]. Dr. G suggested that the proper course at this point would be to proceed with a home palliative care consultation and shift treatment goals to supporting comfort and dignity, as Aaron clearly declined a return to treatment.

During a telemedicine meeting with Dr. G a week later, Aaron asserted, “I don't want to do this for anybody else anymore. It's time to do things only if I want them.” At about this time, Aaron also sought comfort from his therapist and his religious leader, as the prospect of death frightened him, and he was unsure what dying would mean. But he described that being given the choice of what would happen next was empowering – “different, scary, relieving, and right” – in great contrast to repeatedly feeling powerless and demeaned by his many prior chaotic relapses followed by intense pressures to return to treatment. Aaron signed a Do Not Resuscitate (DNR) order and within the next few days was referred to a home palliative and hospice care organization. Dr. G spoke with the organization’s medical director to explain why this brilliant 33-year-old man who was refusing to eat was being referred for palliative care. Aaron hoped that the home palliative care service would help him and his family “process this sorrow and fear.”

Two weeks later, after more than a month of eating nothing and drinking only water, Aaron’s OCD and insomnia were heightened; he worried that by simply smelling his mother’s cooking he might be ingesting those calories. He believed that he might absorb calories from the grocery cart of the person ahead of him at the store. Always reluctant to take medications, he began to consider accepting anxiolytics from the hospice staff, whom he thought were extremely kind.

Aaron noted that by spending no energy forcing himself to eat, he was able to direct energy toward engaging in his faith. His siblings came to visit, and as they talked and laughed, he realized it had been years since they had connected positively. Throughout the course of his eating disorder, every family connection had felt fraught. He summarized his collective family’s response to this pre-terminal phase as, “They were very supportive. They recognize the gravity of this situation. They aren't angry, sad but not fearful.” As he chose to spend his days talking with his parents and sleeping, he noted that he was thinking about others "rather than being so self-absorbed." Imagining his parents’ distress made him sad, and he wanted his parents to keep getting support after his death. "This is one of the hardest things they've had to deal with in their lives."

Even as he rapidly lost weight, Aaron’s body distortions grew worse, and he kept wishing his weight would fall even faster. After almost six weeks without food, Aaron began accepting anxiolytics and antiemetics. He obsessed that someone might have injected his water bottles with calories. When Dr. G asked if he had any words to share for posterity, he expressed words of warning for those who might find themselves in his situation: “OCD will amplify,” “Be prepared for an annoying obsessive brain that might drive you crazy,” and “Just because you aren’t eating doesn’t mean it’s all good now.” He expressed how vital it was to “have people in your life [doctor, parents, family, close friends] whom you trust and can seek reassurance from, who love you unconditionally,” whose comforting words can be “life-saving in terms of giving you peace.” He connected deeply with a feeling that peace comes from God.

After about eight and a half weeks without any food, Aaron was spontaneously vomiting daily and feeling much weaker. Beautifully cared for by home hospice, he began to take low dose morphine for pain and distress. When Aaron’s parents wondered what his death certificate would say about cause of death, Dr. G reassured them that the cause would be anorexia nervosa and malnutrition, not suicide. Often speaking through tears, Aaron’s parents described how they were enjoying a deep loving sweetness with their son that they hadn’t experienced in years, and how they would miss him when he died. They felt compassion for those who lose a loved one abruptly without having time for love, connection, and closure. Often, they saw glimpses of the boy they hadn’t seen in years, as when he looked at photos that made him laugh.

Two weeks later, Aaron passed away with his family surrounding him. Even as they were exhausted and grieving deeply, his parents expressed enormous gratitude for the care he received and for the way they had been able to reconnect with him.

### Case 2: Jessica

Jessica was a 36-year-old woman with a history of OCD and AN, purging subtype (laxatives) that began during her junior year of high school, when she tried to lose weight prior to a vacation. This started a pattern of restricting, binge eating, and then overexercising that persisted into college. When her weight, which had remained normal for some time, did eventually drop, she left college for intensive outpatient eating disorder treatment. It was such a difficult experience that from this time on, she mistrusted eating disorders providers. She lamented that she lost most of the fun of college to her eating disorder.

Due to progressive constipation, Jessica began using laxatives, which led to laxative abuse. She soon found that every time she stopped taking laxatives, her weight skyrocketed (due to rehydration and rebound edema). Ultimately, her AN caused her to drop out of nursing school. Jessica experienced her first hip fracture from severe osteoporosis when she was critically emaciated at age 27, requiring her to move home with her parents; the following month she incurred a stress fracture of her shoulder from using crutches. Her parents pursued guardianship as Jessica was refusing a higher level of care, but her medical team refused to release records to the family’s attorney due to HIPAA. Without the option to pursue guardianship with mandated longer-term residential treatment, her parents came to believe this was the critical juncture where recovery might have been possible, but instead her disease became more entrenched. Over the next 7 months, working with her outpatient primary care provider, dietitian, and therapist, she slowly gained a meaningful amount of weight, although she remained very underweight. Following this, she got an excellent job and once again lived independently from her parents, working productively for three years. However, at age 29, her increased anxiety, the side effects of laxative abuse, and the shame of her anorexia caused her to separate herself from her family and to work from home, increasing her isolation. She checked herself into an expert inpatient medical center to stop using laxatives and then spent a week in inpatient eating disorder treatment before leaving against medical advice. Jessica did manage to stay off laxatives for a year but was plagued by edema. Repeatedly, restriction and overexercise would recur, usually accompanied by laxative abuse, which at its worst consisted of taking 100 tablets a day.

During her initial consultation with Dr. G, Jessica memorably stated, “The eating disorder keeps me out of integrity with my values. It doesn't feel good. You believe something but aren't living it. This is the biggest motivation for wanting to change. I really want to live in alignment with my values, honoring my body, feeling things, stopping being unkind to my body.” Although very kind and compassionate towards others, she struggled to show herself the same grace.

Jessica met criteria for immediate admission back to the inpatient medical service, but given her prior negative experiences with treatment, she wanted to attempt to keep working and live near her parents. She agreed to outpatient care with a multidisciplinary team, focusing on harm-reduction goals. Initially, Jessica was able to follow medical and nutritional recommendations faithfully. Then, within three months of initiating outpatient medical care she fell and sustained a pelvic fracture. This was frightening, disabling, and prevented her from taking her calming (and to her, calorie-burning) nature walks. Overcome by managing the challenges of a rapidly changing body on her own and worried about her fracture and bone health, Jessica readmitted herself to specialized inpatient medical care for medical stabilization. Following stabilization, she agreed to transfer to residential care to attempt a full course of eating disorder treatment. However, after two weeks in the residential care program she left against clinical advice, unable to follow the meal plan consistently and feeling extremely distressed by her bodily changes (even though her weight had barely changed).

At home, Jessica again tried hard to follow treatment recommendations at a harm-reduction level (no laxatives, low caloric intake, gentle movement in the outdoors), but once more the distress of bodily changes was too much for her to bear. About a month after leaving the residential program, Jessica first talked about the possibility of palliative care and began talking with her mom about suicidal thoughts. Most nights she would say she hoped she didn’t wake up the next morning. In order to help Jessica resist the laxatives that gave her such severe abdominal pain and nausea, and still hoping to support her in finding an acceptable degree of harm reduction, Dr. G worked with Jessica to use diuretics to manage fluid weight changes. (Notably, this approach would rarely if ever be offered in a more typical eating disorders treatment plan.) Jessica operated within these guidelines and constraints for the next five months, at times thinking she might be able to persist, but more often lamenting that this strategy was still too difficult and painful. By this point, she had been granted indefinite leave from work and moved in with her parents.

About nine months after initial consultation, Jessica acknowledged that it was time for a palliative approach, confessing, “I’m just ready. It's been a long fight. I'm eating so little, and I'm back on the laxatives every couple of days.” She declined intranasal or intravenous ketamine which might have ameliorated her depression, OCD symptoms, and hopelessness. As she felt progressively miserable physically and psychologically, her suicidality increased. She purchased a gun, and one night she drove to a bridge with thoughts of jumping off, but then decided to return home. She had difficulty finding a therapist who understood terminal AN and who could accept her treatment trajectory, but she found and worked with a kind naturopathic doctor who specialized in mental health, and she did experience some benefit from psychiatric medications.

At this point, fearful of suffering a long, drawn-out death from starvation and unwilling to put her parents through the agony of witnessing this decline, Jessica requested referral to a palliative care specialist who assessed patients for medical aid in dying (MAID). Dr. G spoke with Jessica’s parents repeatedly, assuring them that guardianship and forced treatment were likely now to be futile. The parents had done everything possible to help their daughter find an acceptable quality of life. Jessica signed a DNR order. After speaking with the palliative care physician by phone to discuss the case and advocate for Jessica, Dr. G completed the MAID forms as consulting physician, given that Jessica’s prognosis was presumed to be 6 months or less. The palliative care physician prescribed the MAID medications.

About a year after the initial consultation, and about three months after the MAID consultation, Dr. G saw Jessica for the last time via telemedicine. Jessica wrote to Dr. G in an e-mail, “I’ve been back in a place the last several weeks where the emotional pain and the physical and emotional exhaustion of living like this are just too much for me. I’m trying to make it to the end of May, maybe through June to meet my brother’s upcoming baby before I go.” Jessica described her life as filled with unbearable pain and anxiety. Watching people walking around the neighborhood making future plans felt devastating, because she’d “give anything to be in anybody else’s shoes.” Yet when she thought about stopping diuretics, eating enough food, and gaining weight so she could physically live that life, she said, “it feels impossible.”

Jessica waited several weeks to fill the MAID prescription. She then set multiple dates to use it over a couple of months and changed her mind as that date got closer. A month before her death, she started to receive home hospice services. During this time period, she had long conversations with her parents, brother, and friends, noting that she had many happy memories over her life, apologizing for what she had put them all through over the years, and stating that she hated her eating disorder. She told them she realized that, while her eating disorder behaviors made it seem like she hadn’t loved or trusted them at times, she loved them all very much. She repeatedly told her family that she didn’t want to die, that she didn’t want to miss out on future time with her family, friends, and niece and nephew, but she just couldn’t continue to exist this way. The emotional pain and anxiety were unbearable. She couldn’t live a normal life, and she felt her body was too destroyed to recover. Her parents believe that in her last month she was trying to die naturally by barely eating, reducing her fluid intake, and walking for hours daily, even when she had to sit down often to catch her breath. She stopped driving and carried identification in case she collapsed on a walk. She fainted at home several times in the week before her death, including the night before she died. On the day she took the MAID prescription, she stayed in bed, was at peace, and spent time talking with each parent and her brother. Together as a family, they reminisced, laughed, cried, had their “hug circle” as they had called it since her childhood, and felt surrounded by love. Her parents each held a hand, and her brother sat right next to her. During the three doses of the medicine taken over an hour, she was comfortable and conscious. Within ten minutes of taking the final dose, Jessica closed her eyes, and her breathing slowed.

Jessica didn’t choose to live with anorexia. For all the years she endured living within its prison and myriad complications, her parents ultimately felt strongly that she deserved to choose the time, place, and way of her release. They felt that an unexpected blessing of MAID was that it allowed Jessica to live several months longer than she otherwise would have. Knowing she didn’t have to die a violent death by suicide, that she would have a peaceful way out when the pain and anxiety became unbearable, and that she would be able to die with dignity surrounded by loving family, allowed her to hold on longer. As a dying wish to her mother, she shared, “Mom, I'd like you to do something that will help others not go through what I went through."

### Case 3: Alyssa

Alyssa, the posthumous author on this paper, was a 36-year-old woman with OCD, depression, and restrictive AN who described herself as having “a type A, neurotic personality: a sensitive, compassionate, loving person who's incredibly self-critical and has wanted to do things 0% or 110% with no gray area.” She first felt suicidal at age 13, when she realized that her body was too large to fit into standard dress sizes for her upcoming Bat Mitzvah. She started therapy at that time and was continually in therapy thereafter. After going through high school at a higher weight, the summer before college she vowed to change her body and began exercising in earnest. In college it was easy to restrict. By the time she returned home for Thanksgiving she had lost a substantial amount of weight. Everyone praised her, and she experienced “a deluge of external validation that was irresistible,” firmly establishing her eating disorder by age 18. Alyssa wrestled with AN throughout the rest of her education and career. A brilliant academic, she became the only non-physician Assistant Director of a major academic medical center residency department, mentoring residents and students, doing research, and publishing in major journals.

After struggling with AN for 15 years, during which she received intermittent outpatient support, Alyssa moved in with her parents and reduced her workload. She was extremely helpful in her mother’s struggle with a cancer diagnosis and often underplayed the significance of her own illness. At age 33, to correct severe hypercalcemia she was admitted to the teaching hospital in which she had previously worked. The family felt that her AN was hardly addressed during that hospitalization, in part due to the fact that institutional expertise for AN was confined to a pediatric program. To them, this felt like a vital missed opportunity to attempt changing her disease trajectory, in particular as the only recommendation on discharge was to seek residential eating disorder care.

Alyssa worked for 7 months to obtain insurance authorization for care in a residential eating disorders program, and to gain enough weight to meet their admission criteria. However, upon admission to that program she was deemed still too underweight (by one pound) and was referred to a specialized inpatient medical program. Being rejected for care after so much work also felt like a missed therapeutic opportunity. After a delay, Alyssa spent several weeks in the specialized hospital program and met the minimal criteria for discharge, departing with the understanding that she would immediately enroll in another residential program. However, after discharge from the hospital she refused to do so and could never accept going to an eating disorders program thereafter.

In the years prior to initial consultation with Dr. G, Alyssa’s outpatient treatment team included a local primary care physician with whom she was very close, a therapist she had been seeing regularly in recent years, and an expert eating disorders therapist who had worked with her and the family over the years. Over a period of three years, Alyssa had intermittently thought about and even phoned Dr. G’s outpatient medical clinic, but she never booked an appointment, indicating that she felt very ambivalent about recovery and was considering a palliative care approach. When she finally presented for an initial consultation, Alyssa identified her goals as follows: “I really want a life, to use my Masters in Social Work degree to help others heal, to find a partner, and to experience pleasure, laughter, joy, and freedom, including from my own brain.” As her main barrier she cited the chronic, longstanding shame and body disgust that persistently kept her from meeting her own needs.

At the time of initial consultation, Alyssa met criteria for inpatient medical hospitalization, although she experienced remarkably few physical symptoms, which reinforced her view that she must be “fine.” She declined a higher level of care. Nonetheless, she saw herself as shamefully thin, more keenly felt given her extended family’s experience of the Holocaust. She wanted to be able to walk down the street without turning heads due to being so emaciated, but concurrently struggled to balance this desire against her strong resistance to gaining weight.

Alyssa agreed to ongoing care with the clinic and accepted referral to an expert registered dietitian. She committed to at least attempt a harm reduction approach in which she would slowly restore weight to a point where she could be more physically, mentally, and professionally functional, and where she could resume her yoga practice. However, she stipulated that she would halt weight restoration if and when her AN thinking could no longer bear it. Over the course of the next year or so, she valiantly succeeded in increasing her caloric intake considerably above her previous severely restrictive baseline. But due to the hypermetabolic state often seen in malnourished patients who increase their caloric intake, she experienced no meaningful weight gain.

Nine months after initial consultation, Alyssa emphatically reflected that her goals had not changed, but she had grave doubts about her ability to achieve them. She described feeling “utterly exhausted” and could no longer muster the strength to keep fighting. She vividly described her daily internal battles, struggling every minute of the day to eat enough of her meal plan and constantly fighting against the extreme headwinds of her AN’s resistance. Once she had eaten, she would bitterly berate and punish herself for having done so. At this point she was not certain that her AN was terminal, but she was moving strongly in that direction and wanted to understand her options.

Dr. G clarified that at any time, Alyssa could choose to pursue full recovery and a higher level of care, could continue fighting as she was, or could consider two options that did not focus on recovery. The first option would be choosing palliative care. This would acknowledge that she would likely not survive and also allow her to consider a "bucket list" of experiences for the time she had left. Palliative care would mean that she could eat what appealed to her, with no pressure applied by the team. The treatment focus would be on finding joy and comfort as much as possible. Dr. G emphasized the value of signing a DNR document to protect Alyssa from the mandates of the healthcare system in the event that she experienced an abrupt decline and/or cardiac arrest. Alyssa was also advised that a home palliative care/hospice evaluation would be useful to oversee her treatment as desired during this stage, for emotional and practical support if needed and to protect her parents from any potential legal repercussions should she pass away at home as an emaciated adult. Dr. G noted that for some patients, this stage can last a long time, and that some can “reset” when pressures to gain weight and threats of mandated treatment are removed. In some cases, this state of reduced external pressure might even lead to renewed ability to engage in meaningful harm reduction and even recovery work.

The second option would be to seek hospice care. Hospice care would be suitable if the torments of her AN and the extraordinary difficulties of moving about the world in a skeletal body were beyond being helped by a palliative care approach. Given her faster metabolism, if Alyssa abandoned her attempts to consume a higher meal plan, she would clearly have a less than six-month prognosis and qualify for hospice care. With this option, Dr. G would refer Alyssa to a home hospice service, anticipating that she would become increasingly frail. The home hospice staff would establish warm relationships with Alyssa and her parents, make sure that anxiety, insomnia, nausea, and/or pain were managed, and provide them all access to psychological and spiritual support as desired. During this time, Alyssa could live her life as she chose. As she became less independent, hospice would provide assistive aids such as a shower chair, bedside commode, and hospital bed. The overall goals would be to maximize Alyssa’s comfort, dignity, and time to connect with family.

During this conversation, Dr. G also noted that Alyssa lived in a location where MAID was legal. If she chose the hospice route—and had interest—a referral for the option of MAID was also possible. Alyssa was informed that she herself would have to administer the MAID medications if she chose to use them; no one else could administer them to her. After completing the required regulatory processes and filling the prescription, MAID medications could be used or not as desired. But, as the human body can be exceptionally resilient even with terminal malnutrition, having the medications at hand would give Alyssa the opportunity, while still having an intact brain, to choose not to suffer through additional weeks of extreme physical discomfort and weakness.

A week after these options were reviewed, Alyssa wrote Dr. G:

After deep reflection and discussion with my parents, I’ve decided it makes sense to initiate the Hospice process (Ie evaluation, etc.) now so my family and I are prepared for what may come. I would value your guidance and help with this….I do not know if they have ever worked with patients like myself… I would love for you to be the PCP overseeing this process regardless of the Hospice we select if, and only if, you are comfortable with this. I want to be clear that my priority is to obtain access to the medications that would support my legal right to die should I wind up choosing this path in the future. I feel strongly that based on our thorough discussion, I am aware of my options and their risks and benefits in light of the trajectory of my illness. Please do let me know what I can do to help facilitate initiation of this process. I am available and happy to help.

In a family meeting the following week, Alyssa’s father, a physician, tearfully shared the principles he and Alyssa’s mother had come to accept during intense conversations with their daughter: She had the right to choose care or no care after having been ill for 18 years. There would be no ultimatums. This disease would probably be the reason that "we lose you." They knew how much she had suffered and continued to suffer, and they understood that at some point the psychological anguish would become unbearable for her. They respected that this could be as bad as physical pain. They accepted that when the anguish became unbearable, Alyssa would have the right to end her life by taking medical aid in dying medications. They agreed that financial planning and end of life planning were worthy tasks. To Dr. G and to Alyssa, these words conveyed deeply reassuring love, compassion, and support.

Alyssa’s parents asked whether any treatments remained that might yet change the outcome of her course, specifically noting that Alyssa had not completed a full residential eating disorder program, never fully restored weight, never tried newer psychedelic options such as ketamine, psilocybin, or MDMA, and hadn’t had a feeding tube. Dr. G acknowledged that all but the feeding tube might ordinarily be undertaken prior to someone’s seeking end of life care for AN. Yet, she had been suffering for so long, and despite many conversations about all these treatment possibilities, Alyssa would not consent to any of them. Therefore, given her clarity of understanding around these issues and her sense that she could not fight anymore, everyone had to accept that they weren’t meaningful options. With regards to a surgical feeding tube in the context of AN rather than due an anatomical impediment, Dr. G noted that if someone restricts the “tube God gave them,” i.e. their esophagus, they would also be very likely to restrict through a surgical feeding tube, so that would not be a long term solution.

An excellent home hospice agency agreed to work with Alyssa and her family, and Dr. G placed a referral for a MAID consultation. The palliative care physician met with Alyssa about MAID. Since the idea of requesting MAID for a patient with AN was so foreign and unnerving to him, he asked Alyssa to be assessed formally for decision-making capacity. After a local psychiatrist confirmed that Alyssa clearly possessed decision-making capacity, the palliative care doctor fully accepted Alyssa’s right to enter home hospice care and could understand the rationale for MAID provision. However, even as he and his team provided empathetic support, he ultimately felt personally unable to write the MAID medication prescription due to his discomfort with the unique presentation. Clarification with the state’s Medical Board and other regulatory entities determined that Dr. G, licensed in this state although based in another state, could serve as prescribing physician, and that Alyssa’s longstanding primary care physician could serve as consulting physician. Dr. G prescribed the MAID medications about six weeks after Alyssa entered hospice care. Four days before her death, eager to contribute to this article, Alyssa sent Dr. G the following (unedited) notes about her thoughts on this complicated topic:

Below I share the considerations I made as I weighed the potential benefits and risks of pursing MAID. I share my experience in hopes of offering a first-hand perspective that may help other patients and physicians as they consider and weigh the option of utilizing MAID, rather than offering a prescriptive decision-making tool or recommending that all patients with terminal SEAN have access to such medication.

Personal considerations:

- MAID not pursued in isolation, but rather in the context of being in Hospice care following a terminal dx of anorexia (i.e., estimated 6 months or left to live). I would not have qualified for Hospice care unless my illness was terminal (i.e., not reversible for me in light of physical, mental, emotional damage to my body).
- In my individual case, death was inevitable. I clearly understood my prognosis and accepted this. I saw MAID as an opportunity to select a specified time and circumstances for my death. Death itself is fraught with fear, ambiguity, a sense of powerlessness and tremendous anguish, not just for the patient who is dying, but for that patient’s family. Upon deep reflection, I came to see MAID as an opportunity to relieve my suffering and minimize at least some of my family’s suffering related to my death by choosing the **when** and **how** of my death, rather than “wait” for sudden death from cardiac arrest or other outcome of my illness or experience a slow and protracted death as my family and I watch my body and mind degrade over days and maybe even weeks of time
- I had to ask important questions about my quality of life and whether for me, the quality of my life was more important than the quantity of days I remained alive. I was experiencing extreme physical pain, was unable to walk, could not sit without discomfort, I couldn’t swallow my food, my breath was labored, and I had frequent chest pain. I was not living. I felt like “dead girl barely walking.” For me personally, a longer life spent in bed feeling ill and suffering and dependent on others to provide most of my care was not how I wanted to live. My concerns about this suffering trumped any fear of selecting the route of my death (again, knowing that death was inevitable). Knowing that I could utilize MAID if the suffering became so severe offered me a sense of ease and peace of mind in my final stage of life that I would not have had otherwise
- One question that I needed to answer for myself honestly was whether I understood the impact use of MAID would have on my family. I had to confront that my use of MAID would be difficult for them, not just the idea of my using it but how their presence at the end of my death, watching me administer my own medications to die, would be ingrained in their memories of me as their daughter and their sister, and how this story of my passing would affect my family throughout the generations to come (i.e., what stories would they tell about my life and death, how could this be traumatizing or perhaps seen as healing?). Such questions could only be answered through ongoing involvement and discussion with my family members, which we had with my physicians and amongst ourselves
- Another important question I asked was how would I want a family member to die if I knew their illness was terminal and death was imminent. Would I see their use of MAID as a compassionate act towards themselves? How would I tell their story? Would I extend the compassion I was asking for from them to them if the situation were reversed? I also asked them individually how they would want to die if they could have the option of choosing?
- All in all, a voluntary decision, not made in haste, thoughtful, careful, meticulous. Decision made as arrangements were made for my passing including burial arrangements, financial and family orders.
- Decision also heavily considered with spiritual advisors (chaplain, Rabbi, etc.)

Challenges faced:

- MAID in general is highly controversial and its use is RARE – even for patients who do receive it, many do not end up using it. Only a handful of physicians who support using it. Makes it unknown and scary for physicians and patients alike; limited research
  - Makes acceptance of its use more difficult for family members, too
- Prescribing MAID (for some physicians) may feel counter to physician identity as healer & fixer; may spark deep internal/ethical/moral debate for individual physicians as they weigh the option of whether to prescribe
  - Do they see this as an act of compassion for patients who wish to relieve their suffering?
  - Do they see this as prescribing a means of suicide?
- Anorexia specific – for me, a big issue that caused most ethical debate was whether my case of anorexia nervosa was “reversible.” Many physicians misunderstand SEAN (not even an official DSM diagnosis) and that while anorexia nervosa is a psychiatric illness, it comes with severe medical complications that ultimately are the reason for death. Some of the physicians I worked with could not believe my illness was indeed terminal, but rather felt that there would be something that could be done to reverse the physical damage done to my body that would somehow lengthen my life (even if not for very long – i.e., 1 year).
  - Yes, perhaps I could stay alive for a few months while in the hospital, but I would have to live in the hospital (MDs might see the benefit of this, but could I? NO! This is where my own reflection around quality of life came in)
  - My personal belief that this is what makes having such an extreme form of AN so agonizing – mental and emotional suffering is compounded by painful physical complications
- Gross misunderstanding about anorexia nervosa in general.

Just over a day before she died, Alyssa wrote to Dr. G, “Thank you with all of my heart for helping to make this possible. I view it as a tremendous act of love.” With family and spiritual support surrounding her, Alyssa became unresponsive in the natural course of her malnutrition. Shortly thereafter, she passed away peacefully. She never actually ingested the MAID medication she had at her disposal.

## Discussion

By presenting these three cases, we have intended to convey some of the emotional, moral, and ethical challenges and dilemmas that patients with SE-AN, their families, and their professional caregivers may face at the end of life. Suffering from unrelenting and irredeemable disorders, these patients made difficult choices, ultimately deciding “enough is enough” [18]. The anguish endured by these patients and their families resulted in part from lack of professional understanding and consensus regarding terminal care for patients with AN. Neither the fields of palliative and hospice care nor eating disorders have provided definitions or guidance regarding what constitutes a terminal condition in AN or proper ways to address patients and their families grappling with this condition.

Accordingly, we present the following proposed clinical characteristics of those with terminal AN for consideration by both fields (Table 1). As illustrated by our cases, no set of criteria will apply perfectly to every patient who identifies with having a terminal case of AN. However, based on prior literature on criteria for clinical terminality [15], high SMR in those who have previously received inpatient care, are older, and have a history of more severely medically compromised presentations [6–11], and clinical expertise, the authors propose these clinical characteristics. Some deviation within the second and third characteristics is to be expected and must be individualized to the patient situation. However, the first and fourth must be met in full.

**Table 1:** Proposed clinical characteristics of patients with terminal anorexia nervosa

1. A diagnosis of anorexia nervosa
2. Age of 30 or older
3. Prior persistent engagement in high-quality, multidisciplinary eating disorder care
4. Consistent, clear expression by an individual who possesses decision-making capacity that they understand further treatment to be futile, they choose to stop trying to prolong their lives, and they accept that death will be the natural outcome

## Proposed clinical characteristics of patients with terminal anorexia nervosa

1. **A diagnosis of anorexia nervosa**. Anorexia nervosa is the only eating disorder that carries a guaranteed medical cause of death from malnutrition should weight loss continue unabated. As a result, consistent with literature on duration of life during hunger strikes resulting in death [16], a prognosis of less than 6 months can fairly be established when the patient acknowledges further treatment to be futile and stops engaging in active recovery work. A less than six-month prognosis is congruent with current practice around determination of terminal diagnoses. We fully recognize that patients with SE-AN are likely to have other psychiatric conditions as well.
2. **Age of 30 or older**. This criterion accommodates for what is clinically seen as a potential “late maturation phase” in which even those who have been sick for a long time may discover a shift in values and desires that motivates recovery as they enter their late 20 s. Every effort should be made to promote full recovery and continuation of life in those younger than 30. However, the SMR data of multiple recent studies showing the highest death rates in those with a history of inpatient admissions, longer duration of AN, and age over 30 years old [6–9], taken alongside what functionally has often been a decade or two of exhaustive, ultimately unsuccessful eating disorder treatment, indicates that the age of around 30 as a minimum for terminal AN is reasonable.
3. **Prior persistent engagement in high-quality, multidisciplinary eating disorder care.** Worldwide access to expert eating disorder care varies widely, as does the availability of access to expert inpatient, residential, and full day treatment programs for those with eating disorders. Thus, the definition of care identified here must remain somewhat broad. Before someone can decide they cannot recover, they must have participated in high-quality, expert care to the maximum extent that this is available. This provision should motivate policies that allow for transfers of patients out of designated “networks” that lack expertise, with funding coverage provided at a center of excellence. Ideally, at least some of this treatment will have been undertaken at a sufficiently high level of care to provide extensive structure and support, preferably to the point of full weight restoration at least once in the relatively recent past. Congruent with receipt of such care, qualified health care professionals on the team must support the patient in their decision to stop fighting. We acknowledge that many factors may impact patients’ ability to participate in such care, including lack of access to eating disorders expertise, limitations of the healthcare system, and a personal sense—often based on prior treatment experiences—that admission to certain care settings would cause more harm than good.
4. **Consistent, clear expression by an individual who possesses decision-making capacity that they understand further treatment to be futile, they choose to stop trying to prolong their lives, and they accept that death will be the natural outcome.** Careful determination of decisional capacity is required in each case [19]. An individual who wavers in their conviction or expresses different goals to different people is not yet ready to receive the appellation of terminal AN.

Most eating disorders providers have cared for patients with AN who, despite suffering for decades, continue to show extraordinary determination and resilience. These patients still want help, at least with a harm-avoidance strategy if not with outright full recovery. In these cases, every effort must be made to support the patient’s wishes and provide appropriate resources for recovery. There must be no “giving up” on those who still seek to get better. Indeed, the drive to live and ability to find aspects of life worth fighting for can be seen vividly in the majority of those with AN, even in the face of years or decades of illness and suffering. The psychological imperatives of AN that often lead patients to resist or refuse clinically appropriate care, hazarding medical and psychological risk and deterioration, may seem to conflict with a stated desire to keep trying for recovery. However, in honoring patient autonomy, responsive care must always be offered as long as an individual states that this is their wish.

Patients in their earlier and younger years of AN may say they would rather die than gain weight or nourish themselves properly, a characteristic indicating that AN may present as an ego-syntonic mental illness. Nonetheless, the majority of patients with AN ultimately recover, and such expressions of anguish can be met with compassion and appropriate multidisciplinary care. We would not condone accepting a terminal diagnosis in younger patients. Of note, there are no explicit physiologic markers or measurables (weight, degree of weight loss, presence of or degree of organ failure, vital signs) which delineate someone with terminal AN. Even individuals with extreme medical malnutrition may recover fully if they so choose and have access to expert care. By contrast, if all criteria for terminal AN are met, as in the case of Aaron, individuals should not be obliged to demonstrate extreme medical instability before having the right to choose to stop fighting. Furthermore, while the obsessional ruminations of individuals with AN can be perplexing, clinicians should not regard the presence of body distortions and food fears as proof that these patients are unable to understand personal options and make reasoned health care decisions.

How can we determine that patients with severe anorexia nervosa possess the clinical decision-making capacity necessary to permit them to withdraw from treatment? With respect to decision-making capacity, four traditional criteria are usually applied: understanding, appreciation, ability to reason, and communication of decision [20]. In Dr. G’s estimation, confirmed in the two cases where formal independent assessment by a psychiatrist was performed, each of the patients met these criteria and was therefore capable of deciding to withdraw from conventional treatment. Alyssa’s clear, incisive writing just days before her death beautifully illustrates the insight and cognitive capacity that many patients with AN possess right up to the end of their life.

Clinical, legal, and ethical commentators in the field concur that withdrawal from treatment may be appropriate when further treatment, whether voluntary of involuntary, will provide only brief improvement, and is unlikely to offer sustained quality of life [21, 22]. A formal assessment of decision-making capacity may help ameliorate family member fears that such an important decision is being made in an appropriate and ethical manner, especially when AN fears and distortions can seem so irrational. In addition, a formal bioethics evaluation might be valuable, but consideration of this must be balanced against most bioethicists’ lack of experience with patients who have AN, with the risk that their own innate and misguided reaction that “this patient just has to eat” could undermine a qualified patient’s decisions that are supported by their longstanding care team and family. Even medical ethicists must be wary about how their own cognitive and affective biases might influence their recommendations. [23]

Family members and carers play an immensely important role in the lives of those with AN. They bear witness to the suffering and challenges experienced by those with AN and are usually directly involved in the recovery process in multiple ways (financial/material support/behavioral support/engagement in the therapeutic work, among others). Many dread the day their child legally becomes an adult and can choose to exclude them from the details of recovery work, such that they become the financial supporters of care they are no longer privy to. The exhaustion, fear, love, and hope experienced by family members cannot be overstated. In any case where a patient meets the criteria for terminal AN, it is always preferable to include family members in the discussions and ideally come to a consensus. There may be dissent within a family about whether their loved one should be allowed to make the decision to stop fighting. These three cases illustrated how each family was meaningfully involved in the clinical discussions in the months before each patient’s death. Each family’s ultimate acceptance (through deep grief) of their son or daughter’s prognosis and choice contributed to a heightened sense of connection and love prior to death.

Acknowledging the considerable controversies surrounding MAID for patients with mental disorders [24–26], we also submit that patients with terminal AN who are severely physiologically compromised, and whose end-of life suffering results from both psychological and physical pain, should be afforded access to medical aid in dying in locations where such assistance has been legalized—just like other patients with terminal conditions.

## Conclusions

AN confers an exceptionally high death rate. The lack of acceptance of terminality in AN and the absence of professionally condoned protocols and standard procedures for supporting patients and families through these phases further complicates end-of-life stages for the adults with AN who cannot keep fighting. These represent a small fraction even of the population of those with SE-AN. Per our proposed clinical characteristics, patients must not only decline further recovery-oriented treatment (which is not uncommon at times for those with AN), but also must explicitly and consistently choose to stop trying to prolong their lives, accepting that death will be the natural outcome. When a patient begins talking about the possibility of not being able to survive, every effort should be made to validate such a serious perspective and to offer an individualized and thoughtful series of harm reduction strategies and treatment options that might make life bearable. However, the process of seeking alternatives to death must not be so exhaustive as to disrespect limits the patient sets; while a family might be desperate for their loved one to try an experimental treatment or “just try going to treatment one more time,” they must ultimately accept the patient’s lack of consent for these.

Our proposed clinical characteristics of patients with terminal AN have no bearing on those who wish to keep fighting despite very long-standing and severe disease, even when their eating disorder behaviors seem incongruent with survival. Very specifically, to move toward a designation of terminal AN, an individual must express consistently that they can no longer live with their disease and will no longer maintain a minimum nutritional intake needed to support life. To be clear, each patient is unique and requires careful individual assessment and consideration as to the best approach going forward. Consistent with calls from others regarding the need for better definition and agreement regarding labeling and staging for SE-AN in general [1,2,3,4 5], the authors hope that these cases and characteristics of those with terminal AN will provide a starting point for identification, care, and further discussion. We would strongly encourage the development of expert consensus criteria and clinical guidelines endorsed by both the fields of palliative and hospice care and eating disorders. These brave, suffering individuals deserve no less.

## Data Availability

All narrative data and record of e-mails exchanged with patients and families throughout and after their care with the clinic are available.

## Abbreviations

AN: Anorexia nervosa
Dr. G: Dr. Jennifer Gaudiani
MAID: Medical Aid in Dying
OCD: Obsessive Compulsive Disorder
SE-AN: Severe and enduring anorexia nervosa
